# Temperature Dependence of Metals Accumulation and Removal Kinetics by *Arabidopsis halleri* ssp. *gemmifera*

**DOI:** 10.3390/plants12040877

**Published:** 2023-02-15

**Authors:** Hiroshi Kudo, Zhaojie Qian, Chihiro Inoue, Mei-Fang Chien

**Affiliations:** Graduate School of Environmental Studies, Tohoku University, Sendai 980-8579, Japan

**Keywords:** *Arabidopsis halleri* ssp. *gemmifera*, heavy metal, removal kinetics, temperature dependence

## Abstract

Cadmium (Cd), which is present in zinc (Zn) ore, is a toxic metal and causes contamination globally. Phytoremediation is a promising technology for the remediation of sites with low and moderate contamination. Temperature is an important factor in phytoremediation because it has an impact on both plant biomass and the accumulation of heavy metals. However, little is known about the influence of temperature on heavy metal accumulation by the Cd and Zn hyperaccumulator *Arabidopsis halleri* ssp. *gemmifera*. The effect of temperature on the distribution of Cd and Zn in *A. halleri* ssp. *gemmifera* and the mechanism of metal removal from solution were investigated in this study. Our results showed that the temperature dependence of the distribution of Cd and Zn in the plant was different, which may suggest that the mechanisms of xylem loading were different between Cd and Zn. Although Cd and Zn have partially similar transport pathways, the removal kinetics based on the first-order reaction rate constant revealed that the temperature which maximized rate of absorption was different between Cd and Zn. This study suggests a potential for efficient Cd phytoextraction using *A. halleri* ssp *gemmifera* in Cd and Zn co-existing environments.

## 1. Introduction

Cadmium (Cd), a non-essential element for humans and plants, is one of the most toxic heavy metals in soil and water. Human activities, including mining for zinc (Zn) [1,2], applying phosphate fertilizers containing Cd impurities, and sewage sludge [3], are sources of Cd contamination in soil. According to reports, 7% of China’s farmland exceeds the Cd limit of 0.3 mg/kg set by the Ministry of Environmental Protection [4]. As rice accumulates Cd readily, Asian people who consume rice as a staple diet are concerned about the risk of Cd to their health [5]. Long-term Cd intake results in chronic poisoning symptoms, including renal failure, osteoporosis, and Itai-Itai disease at worst [6,7]. The zinc (Zn) concentration tends to be high in Cd-contaminated soil due to mining, and smelting. Although Zn is an essential element for humans, the excessive intake of Zn causes diarrhea and metal fume fever, and may increase the risk of prostate cancer [8].

Phytoremediation is a technique for cleaning up the environment by using plants’ biological processes. Its technological subdivisions include phytoextraction, phytofiltration, phytostabilization, phytovolatilization, and phytodegradation [9]. Among these, phytoextraction, using plants to extract and remove contaminants from soil or water, is a sustainable technique to remediate low to moderately contaminated environments. This method has a lower cost and environmental impact than conventional physicochemical remediation strategies [10]. Regarding phytoextraction, hyperaccumulator plants that can accumulate contaminants in their shoots to a greater extent are often used. A potential plant for phytoremediation is *Arabidopsis halleri* ssp. *gemmifera*. This plant has been identified as a hyperaccumulator that can accumulate Cd and Zn up to 5641 mg/kg and 26,400 mg/kg in its shoots, respectively [11,12]. This plant’s range includes Japan, Korea, north-eastern China, and the Russian Far East, including Sakhalin and Kamchatka [13]. This plant also has the ability to tolerate extremely low temperatures up to −17.5 °C during field cultivation [14].

Various environmental factors are responsible for affecting the efficiency of phytoextraction. In particular, temperature is a key factor for phytoextraction because it affects both biomass and the accumulation of metals. It has been reported that plant biomass increases with increases in temperature up to an optimum temperature, then decreases thereafter [15,16]. Previous studies have reported that Cd uptake by *Peltigera horizontalis* and *Dumortiera hirsuta* is stimulated by increasing temperatures [17,18]. On the other hand, arsenic (As) accumulation in the shoots of *Pteris cretica* does not increase with increasing temperatures [19]. In the case of *A. halleri* ssp. *gemmifera*, there are only a few reports available on the accumulation of Cd and Zn at different temperatures, although understanding the influence of temperature on heavy metal accumulation may contribute to the practical application of phytoextraction. Therefore, in this study, the effect of temperature on the accumulation and removal kinetics of Cd and Zn by *A. halleri* ssp. *gemmifera* was investigated to provide necessary information for efficient phytoextraction.

## 2. Results and Discussion

### 2.1. Plant Biomass and Heavy Metal Accumulation at Different Temperatures

**Figure 1:**
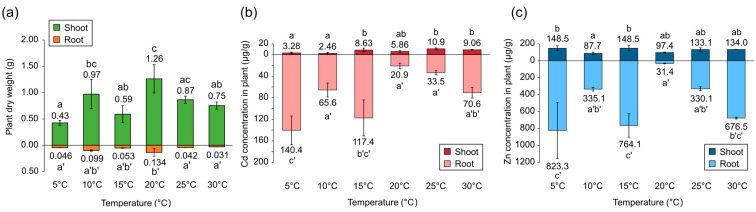
(**a**) Plant dry weights of *A. halleri* ssp. *gemmifera,* (**b**) Cd concentration in plants, and (**c**) Zn concentration in plants. The same letters indicate no significant differences between samples according to the Tukey–Kramer test (*p* < 0.05).

**Figure 2:**
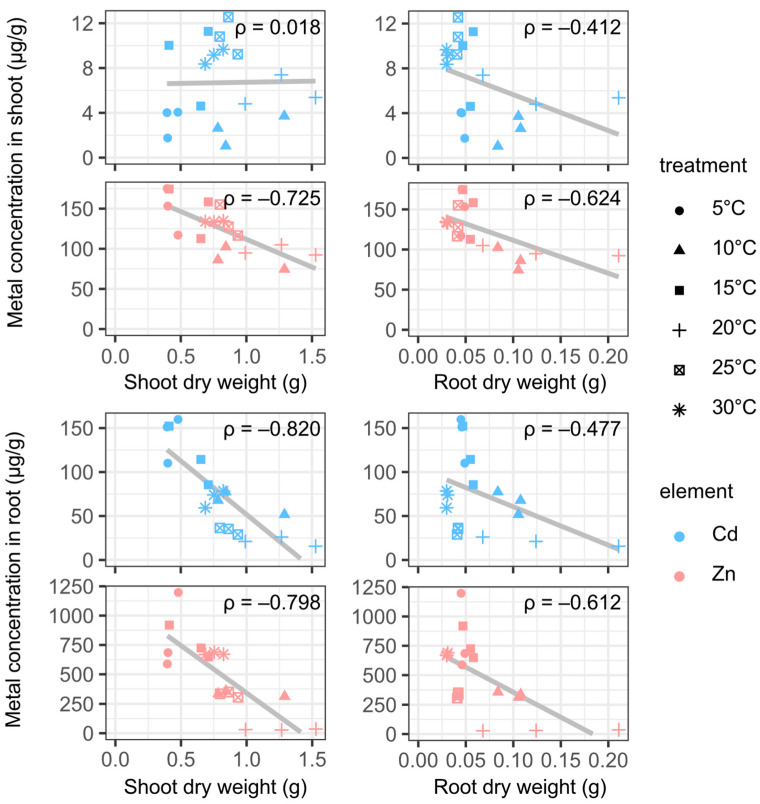
The correlation between the plant dry weight and metal concentration in the plant. ρ represents the Pearson correlation coefficient.

The changes in the biomass and concentration of metals in plants under different temperatures were determined. Plants with similar appearances (age, number of leaves, and height) were chosen for this experiment, as it would be difficult to compare the dry weight of the same plant before and after the experiment. The biomass of *A. halleri* ssp. *gemmifera* at the end of the experiment was significantly different (Figure 1a) and seemed to have an impact on the concentration of Cd and Zn in the plants (Figure 1b,c). In fact, with the exception of the Cd concentration in the shoots, the metal concentrations in plants showed a high correlation with biomass (Figure 2). While the Cd concentrations in shoots were not correlated with shoot biomass, they showed a moderate correlation with root biomass. Van Belleghem et al. reported that the cortical apoplast of *Arabidopsis thaliana* was an early sequestering site of Cd in the root in 1 μM Cd-exposure experiments [20]. Furthermore, in the case of *Arabidopsis halleri* ssp. *halleri*, Cd was accumulated in root epidermal cell walls under 100 μM Cd exposure [21]. Hence, the correlation between the root biomass and Cd concentration in shoots may suggest that the accumulation of Cd in roots may contribute to the short-term control of the Cd concentrations in shoots.

The Cd concentrations in plants ranged from 7.9 μg to 11.1 μg and the Zn concentrations ranged from 100.3 μg to 128.0 μg, respectively (Table 1). The Cd and Zn added to the solution were almost absorbed by the plants in all of the treatments. Whereas the Cd and Zn concentrations in the 5°C treatment appeared to be lower than those at the other temperatures, there was no significant difference in the total metal ions accumulated in the plants. Therefore, it was anticipated that plant biomass had no influence on the amount of metal accumulation.

**Figure 3:**
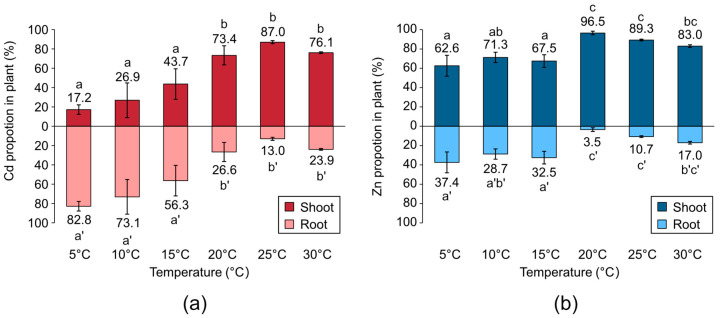
(**a**) Cd and (**b**) Zn distributions in the plant at different temperatures. The same letters indicate no significant differences between samples according to the Tukey–Kramer test (*p* < 0.05).

Regarding the distribution of the metals in the plants, Figure 3 shows that, under warm temperatures (20–30 °C), both Cd and Zn were more evenly distributed in the shoots than under cold conditions (5–15 °C). Note that the distribution of Cd in shoots and roots showed a temperature-dependent change from 5 °C to 25 °C (Figure 3a). Meanwhile, in the case of Zn, the temperature dependency was unclear (Figure 3b). The temperature dependence of the Cd distribution suggests that heavy-metal ATPase 2 (HMA2) and HMA4 were primarily responsible for moving Cd from the roots to the shoots. HMA2, HMA4, and Plant Cadmium Resistance 2 (PCR2) are known as Cd/Zn transporters in *Arabidopsis* that are engaged in xylem loading and help to move metals from the roots to the shoots [22,23,24]. Although the transport mechanism of PCR2 is still unknown, HMA2 and HMA4 are both P_1B_-type ATPases that require energy produced by ATP hydrolysis to transport the metal ions. ATP generation in plants is governed by photosynthesis, and the rate of photosynthesis increases to an optimal level along with temperature before decreasing [25]. Although cold-adapted plants that are active in early spring, such as *A. halleri* ssp. *gemmiferam* can photosynthesize between 0 and 30 °C without harm [26], the conditions that maximize the rate of photosynthesis depend on the light intensity, CO_2_ concentration, and plant species [27]. In this study, the Cd distribution increased with increasing temperature up to 25 °C and then decreased. The temperature-dependence behavior of the Cd distribution was similar to that of photosynthesis, which may suggest that HMA transporter, which is dependent on ATP, contributed to the temperature dependence of the Cd distribution in plant shoots. Actually, Ueno et al. reported that Cd transfer from the root medium to the xylem in *A. halleri* ssp. *halleri* was an energy-dependent process because the uncoupling agent that inhibited ATP synthesis inhibited only Cd transfer from the root medium to the xylem without inhibiting transfer from the solution to the root [28]. Moreover, in the case of *A. thaliana*, HMA2 and HMA4 were reported to be the major mechanisms for root-to-shoot Cd translocation [24].

Despite the fact that HMA2 and HMA4 can also transport Zn, the distribution of Zn in plants did not clearly show a temperature dependency. This suggests that, in addition to HMA2 and HMA4, Zn was translocated from roots to shoots via other pathways, including PCR2, or by mechanisms different from Cd, as suggested by Wiyono et al. [29]. For instance, Cornu et al. suggested that transporters that can transport metal–nicotianamine complexes are involved in the xylem loading of Zn–nicotianamine complexes in *A. halleri* ssp. *halleri* [30]. Alternately, FRD3, which effluxes citrate into the vascular tissues of the roots, may regulate Zn translocation in plants [31]. According to reports, exposure to Cd suppresses the expression of AtFRD3 [32], and the knockout mutant of AtFRD3 over-accumulates Zn in the leaves [33]. Hence, Zn translocation may be partly regulated by FRD3 when Cd and Zn coexist in roots. As Zn is an essential nutrient involved in a wide variety of physiological processes [34], it may be preferentially translocated to shoots by such mechanisms while reducing the influence of Cd.

### 2.2. Removal Kinetics of Metals Ions from Solution

**Figure 4:**
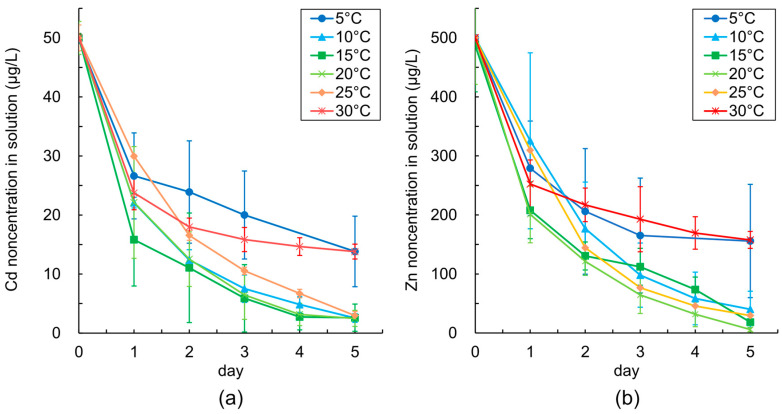
Changes in the (**a**) Cd concentrations and (**b**) Zn concentrations in the solutions under different temperatures.

Regardless of temperature, the Cd and Zn concentrations in solutions decreased to half in a single day (Figure 4). Meanwhile, from day 2, the change in the metal concentration slowed down in 5 °C and 30 °C treatments, and the final metal concentrations of both metals were higher than those in other temperature treatments. The final Cd concentrations in solutions were lower than 3.0 μg/L in the 10–25 °C treatments and approximately 14 μg/L in the 5 °C and 30 °C treatments (Figure 4a). The difference in the final Cd concentration was statistically significant (*p* < 0.05). The final Zn concentration was lower than 40 μg/L in the 10–25 °C treatments and approximately 160 μg/L in the 5–30 °C treatments (Figure 4b). The final Zn concentration between the treatments at 30° C and 10–25 °C varied significantly (*p* < 0.05). 

**Figure 5:**
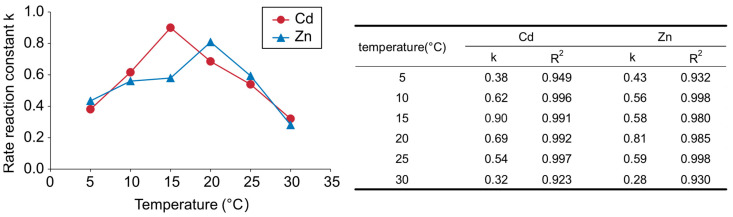
Change in the reaction rate constant for Cd and Zn removal from solution by *A. halleri* ssp. *gemmifera*.

In this study, the uptake behavior of metals by the plants was assumed to be a first-order reaction. The first-order reaction rate constants were estimated using the metal ion concentrations in solutions using Equation (1). As shown in Figure 5, although the R^2^ values between the 5–30 °C treatments were lower than those of the other treatments, the Cd and Zn removal kinetics well-fitted a first-order reaction model between 10 and 25 °C. Thus, the reaction of heavy metal uptake from the solution to the plants was generally simulated by a first-order reaction model. When the temperature rose from 5 °C to 15 °C, the rate constants of Cd increased; however, as the temperature rose above 15 °C, they decreased. Meanwhile, the rate constant of Zn was highest at 20 °C. The coefficient of determination was lower in the 5 °C and 30 °C treatments compared with the other conditions. The fact that the metal uptake mechanism could not fit the first-order reaction model suggested that it was a more complicated reaction. This result suggested that *A. halleri* ssp. *gemmifera* released metal ions from its roots due to temperature stress. Cd/Zn transporter PCR2 is expressed in the epidermis of root cells to efflux excess heavy metals [20]. Although it is unknown whether transporter expression changes in response to temperature, temperature stress-induced heavy metal efflux has been seen in other hyperaccumulator plants. For instance, three *Pteris* Arsenic (As) hyperaccumulators were reported to release As from their root at 5 °C treatment, even though *Pteris cretica*, *Pteris multifida*, and *A. halleri* ssp. *gemmifera* are cold-tolerant [19].

The differences in the ratio of the metal concentration in the shoots to that in the roots between Cd and Zn (Table 1) and differences in the metal distribution in plants (Figure 3) suggested that the transport rates for Cd were different from those of Zn. Nevertheless, the maximum rate constant of Cd and Zn was generally similar (Figure 5). These results suggest that the rate-determining processes of Cd and Zn uptake were the processes before xylem loading, such as solution-to-root translocation or uptake and remobilization by root vacuoles. In addition, the temperatures that elevated the rate constants of Cd and Zn were different. Both Cd and Zn enter the root system through specific membrane transporters, mainly ZRT/IRT-like protein (ZIP) transporters [35]. To date, a total of 18 ZIP transporters have been annotated in *A. thaliana* [36]. A study on 15 ZIP genes that are phylogenetically close to *AtIRT1* reported that the expression of *AtIRT1*, *AtIRT2*, and *AtZIP9* in roots was increased by Cd exposure, and only *AtIRT1* was found to be responsible for Cd accumulation [37]. Although the roles of other *Arabidopsis* ZIP proteins for Zn uptake have not been fully explored, Zn is also transported by IRT1 [35]. As Cd and Zn may coexist in the same environment without competing for the same transport pathways, it is worth noting that the temperatures that maximized Cd and Zn absorption were varied. The transporters involved in the transport of metals to root vacuoles are different for Cd and Zn [38,39]. This may be a factor that contributed to the differences in the temperature that maximized the rate of metal absorption. In addition, metal efflux by PCR2 and the temperature-dependent activity of transporter proteins may influence the rate of metal uptake. However, little is known about the influence of temperature on transporter activity. Future studies on the effect of temperature on transporters may lead to efficient Cd phytoextraction in Cd and Zn co-existing environments.

## 3. Materials and Methods

### 3.1. Plant Cultivation

The seeds of *A. halleri* ssp. *gemmifera* were sown in a plastic tray filled with 0.2-strength Hoagland solution. The Hoagland solution was composed of 8 mM KNO_3_, 4 mM Ca(NO_3_)_2_, 2 mM MgSO_4_, 1 mM (NH_4_)(H_2_PO_4_), 50 μM H_3_BO_3_, 9 μM MnSO_4_, 1 μM ZnSO_4_, 0.2 μM CuSO_4_, 0.1 μM Na_2_MoO_4_, and 60 μM Fe(III)-EDTA. After one month of seed germination, the seedlings were transferred to 250 mL plastic bottles with 0.2-strength Hoagland solution. Each bottle contained one seedling and 200 mL of the solution. The basal nutrient solution was replaced once a week. All seedlings were grown in a growth chamber (Biotron LH-241SP, Nippon Medical & Chemical Instruments Co., Ltd., Osaka, Japan) under the following conditions: 72.9 µmol m^−2^ s^−1^ photon flux density supplied by a cool white fluorescent lamp, 70% humidity, 20 °C, and a 16 h light and 8 h dark photoperiod. After 2~3 months of precultivation in the growth chamber, healthy plants with similar shoot and root sizes were selected and used for further experiments.

### 3.2. Heavy Metals Removal Experiments under Different Temperatures

The experiment was conducted according to our previous study [40]. Two–three-month-old *A. halleri* ssp. *gemmifera* seedlings were thoroughly cleaned under running tap water to remove sediments and other particles, and finally rinsed with Milli-Q water. After that, the seedlings were placed in a container with 200 mL of Hoagland solution at 0.2 strength. The initial concentrations of heavy metals in the shoots were 0.04 mg/kg Cd and 19.2 mg/kg Zn, respectively (mean of 3 plants). The initial metal amounts in plants were Cd 0.07 ± 0.04 μg/plant and Zn 18.0 ± 5.7 μg/plant. The plants were cultivated at six different temperatures (5 °C, 10 °C, 15 °C, 20 °C, 25 °C, and 30 °C) in growth chambers. Three replicates for each treatment were used, and Hoagland solution was exchanged every day. After 3 days of temperature acclimation, Cd(NO_3_)_2_ and Zn(NO_3_)_2_ were added to all replicates independently to reach Cd and Zn concentrations of 50 μg/L, and 500 μg/L, respectively. On each day of the experiment, 2 mL of the solution was taken, and it was completed after 5 days. This experiment focused on the effect of temperature on the absorption kinetics of metals. The concentration of Zn in Hoagland solution was lower compared with the Zn addition treatment and was assumed to be absorbed in a short period of time. Hence, no control was set up as it was difficult to compare the results between the control and the other treatments. All plants were rinsed twice in Milli-Q water for half a minute each. They were dried at 80 °C for a week and then weighed to determine the plant dry weight. All dried samples were digested in 70% HNO_3_ by heating at 130 °C. Inductively coupled plasma mass spectrometry (ICP-MS; ELAN 9000, Perkin Elmer, Inc., Waltham, MA, USA) was used for the quantitative analysis of the Cd and Zn concentrations. Indium was added at 10 μg/L to each sample as ab internal standard. The measurements were taken under the following conditions: nebulizer gas flow: 0.87 L/min; auxiliary gas flow: 1.2 L/min; plasma gas flow: 16 L/min; lens voltage: 9.0 V; and ICP RF power: 1100 W. Readings of each sample were taken in triplicate.

### 3.3. Removal Kinetics of Metals from Solution

The rate of a chemical reaction can provide important information regarding the mechanism and behavior at which biological interactions occur in living systems [41]. In order to assess the rate of Cd and Zn removal from the solution, the first-order reaction model was used. The linear form of the first-order equation is expressed as follows:

(1) $$\begin{array}{c} \ln\frac{C_{t}}{C_{0}}=-kt \end{array}$$

where C_t_ is the metal concentration in solution (μg/L) at time t (day), C_0_ is the initial metal concentration in solution (μg/L), and k is the first-order reaction rate constant. The coefficient of determination (R^2^) was obtained from the linear regression line between the rate constant of k and ln(C_t_/C_0_).

### 3.4. Statistical Analysis

The average of three replicates was used for every data, along with the standard error of the mean. Statistical significance was determined by the Tukey–Kramer method using the multicomp package in R version 4.0.5 [42].

## 4. Conclusions

In this work, we demonstrated that, in a short-term experiment, the temperature dependency of the distribution of Cd and Zn in *A. halleri* ssp. *gemmifera* was different. The translocation of Cd from the roots to the shoot was clearly temperature-dependent and fastest under the 25 °C treatment. This result suggests that Cd xylem loading was an energy-dependent process via HMA transporters. On the other hand, the Zn distribution did not show temperature dependency, suggesting that Zn was partly transferred to the xylem via metal–nicotianamine transporters, or through the mechanism involving citrate transporter FRD3. The removal behavior based on the first-order reaction model suggested that the processes before xylem loading determined the rate of metal uptake, and the maximum rates of metal uptake were different for Cd and Zn. Although further study on the influence of temperature on metal transporter proteins is needed, this study suggested the Cd phytoextraction potential of *A. halleri* ssp. *gemmifera* in Cd and Zn co-existing environments.

## Figures and Tables

**Table 1:** Translocation factor and total metal amount in plants. The same letters indicate no significant differences between samples according to the Tukey–Kramer test (*p* < 0.05).

Temperature (°C)	Translocation Factor		Total Metal Amount in Plant (μg)	
	Cd	Zn	Cd	Zn
5	0.02 ± 0.01 ^a^	0.21 ± 0.10 ^a^	7.9 ± 1.6 ^a^	100.3 ± 8.6 ^a^
10	0.04 ± 0.03 ^ab^	0.26 ± 0.02 ^a^	9.0 ± 1.5 ^a^	116.3 ± 12.7 ^a^
15	0.08 ± 0.05 ^ab^	0.20 ± 0.04 ^a^	11.1 ± 1.9 ^a^	125.9 ± 20.9 ^a^
20	0.28 ± 0.06 ^c^	3.15 ± 0.62 ^b^	10.0 ± 2.3 ^a^	127.1 ± 26.3 ^a^
25	0.32 ± 0.03 ^c^	0.40 ± 0.05 ^a^	10.8 ± 1.4 ^a^	128.0 ± 8.5 ^a^
30	0.13 ± 0.01 ^b^	0.20 ± 0.00 ^a^	9.0 ± 1.4 ^a^	121.8 ± 9.6 ^a^

## Data Availability

Not applicable.

## References

[1] Zhang X., Yang L., Li Y., Li H., Wang W., Ye B. (2012). Impacts of lead/zinc mining and smelting on the environment and human health in China. Environ. Monit. Assess..

[2] Zhou Z., Chen Z., Pan H., Sun B., Zeng D., He L., Yang R., Zhou G. (2018). Cadmium contamination in soils and crops in four mining areas, China. J. Geochem. Explor..

[3] Bigalke M., Ulrich A., Rehmus A., Keller A. (2017). Accumulation of cadmium and uranium in arable soils in Switzerland. Environ. Pollut..

[4] Zhao F.J., Ma Y., Zhu Y.G., Tang Z., McGrath S.P. (2015). Soil contamination in China: Current status and mitigation strategies. Environ. Sci. Technol..

[5] Meharg A.A., Norton G., Deacon C., Williams P., Adomako E.E., Price A., Zhu Y., Li G., Zhao F.J., McGrath S. (2013). Variation in rice cadmium related to human exposure. Environ. Sci. Technol..

[6] Inaba T., Kobayashi E., Suwazono Y., Uetani M., Oishi M., Nakagawa H., Nogawa K. (2005). Estimation of cumulative cadmium intake causing Itai-itai disease. Toxicol. Lett..

[7] Bertin G., Averbeck D. (2006). Cadmium: Cellular effects, modifications of biomolecules, modulation of DNA repair and genotoxic consequences (a review). Biochimie.

[8] Plum L.M., Rink L., Haase H. (2010). The essential toxin: Impact of zinc on human health. Int. J. Environ. Res. Public Health.

[9] Yan A., Wang Y., Tan S.N., Mohd Yusof M.L., Ghosh S., Chen Z. (2020). Phytoremediation: A promising approach for revegetation of heavy metal-polluted land. Front. Plant Sci..

[10] Lee J.H. (2013). An overview of phytoremediation as a potentially promising technology for environmental pollution control. Biotechnol. Bioprocess Eng..

[11] Kashem M.A., Singh B.R., Kubota H., Nagashima R.S., Kitajima N., Kondo T., Kawai S. (2007). Assessing the potential of *Arabidopsis halleri* ssp. *gemmifera* as a new cadmium hyperaccumulator grown in hydroponics. Can. J. Plant Sci..

[12] Kashem M.A., Singh B.R., Kubota H., Sugawara R., Kitajima N., Kondo T., Kawai S. (2010). Zinc tolerance and uptake by *Arabidopsis halleri* ssp. *gemmifera* grown in nutrient solution. Environ. Sci. Pollut. Res..

[13] Honjo M.N., Kudoh H. (2019). *Arabidopsis halleri*: A perennial model system for studying population differentiation and local adaptation. AoB Plants.

[14] Zhang Z., Wen X., Huang Y., Inoue C., Liang Y. (2017). Higher accumulation capacity of cadmium than zinc by *Arabidopsis halleri* ssp. *germmifera* in the field using different sowing strategies. Plant Soil.

[15] Gajanayake B., Reddy K.R., Shankle M.W., Arabcibia R.A., Villordon A.O. (2014). Quantifying storage root initiation, growth, and developmental responses of sweetpotato to early season temperature. Agron. J..

[16] Walne C.H., Reddy K.R. (2022). Temperature effects on the shoot and root growth, development, and biomass accumulation of corn (*Zea mays* L.). Agriculture.

[17] Beckett R.P., Brown D.H. (1984). The control of cadmium uptake in the lichen genus *peltigera*. J. Exp. Bot..

[18] Mautsoe P.J., Beckett R.P. (1996). A preliminary study of the factors affecting the kinetics of cadmium uptake by the liverwort *Dumortiera hirsuta*. S. Afr. J. Bot..

[19] Rahman F., Sugawara K., Wei S., Kohda Y.H., Chien M.F., Inoue C. (2021). Influence of low temperature on comparative arsenic accumulation and release by three *Pteris* hyperaccumulators. J. Environ. Sci. Health Part A.

[20] Van Belleghem F., Cuypers A., Semane B., Smeets K., Vangronsveld J., D’Haen J., Valcke R. (2007). Subcellular localization of cadmium in roots and leaves of *Arabidopsis thaliana*. New Phytol..

[21] Küpper H., Lombi E., Zhao F., McGrath S.P. (2000). Cellular compartmentation of cadmium and zinc in relation to other elements in the hyperaccumulator *Arabidopsis halleri*. Planta.

[22] Song W.Y., Choi K.S., Kim D.Y., Geisler M., Park J., Vincenzetti V., Schellenberg M., Kim S.H., Lim Y.P., Noh E.W. (2010). *Arabidopsis* PCR2 is a zinc exporter involved in both zinc extrusion and long-distance zinc transport. Plant Cell.

[23] Verret F., Gravot A., Auroy P., Leonhardt N., David P., Nussaume L., Vavasseur A., Richaud P. (2004). Overexpression of AtHMA4 enhances root-to-shoot translocation of zinc and cadmium and plant metal tolerance. FEBS Lett..

[24] Wong C.K.E., Cobbett C.S. (2009). HMA P-type ATPases are the major mechanism for root-to-shoot Cd translocation in *Arabidopsis thaliana*. New Phytol..

[25] Crous K.Y., Uddling J., De Kauwe M.G. (2022). Temperature responses of photosynthesis and respiration in evergreen trees from boreal to tropical latitudes. New Phytol..

[26] Sage R.F., Kubien D.S. (2007). The temperature response of C3 and C4 photosynthesis. Plant Cell Environ..

[27] Hikosaka K., Ishikawa K., Borjigidai A., Muller O., Onoda Y. (2006). Temperature acclimation of photosynthesis: Mechanisms involved in the changes in temperature dependence of photosynthetic rate. J. Exp. Bot..

[28] Ueno D., Iwashita T., Zhao F.J., Ma J.F. (2008). Characterization of Cd translocation and identification of the Cd form in xylem sap of the Cd-hyperaccumulator *Arabidopsis halleri*. Plant Cell Physiol..

[29] Wiyono C.D.A.P., Inoue C., Chien M.F. (2021). *HMA4* and *IRT3* as indicators accounting for different responses to Cd and Zn by hyperaccumulator *Arabidopsis helleri* ssp. gemmifera. Plant Stress.

[30] Cornu J.Y., Deinlein U., Höreth S., Braun M., Schmidt H., Weber M., Persson D.P., Husted S., Schjoerring J.K., Clemens S. (2015). Contrasting effects of nicotianamine synthase knockdown on zinc and nickel tolerance and accumulation in the zinc/cadmium hyperaccumulator *Arabidopsis halleri*. New Phytol..

[31] Verbruggen N., Hermans C., Schat H. (2009). Molecular mechanisms of metal hyperaccumulation in plants. New Phytol..

[32] Van De Mortel J.E., Schat H., Moerland P.D., Van Themaat E.V.L., Van Der Ent S., Blankestijn H., Ghandilyan A., Tsiatsiani S., Aarts M.G.M. (2008). Expression differences for genes involved in lignin, glutathione and sulphate metabolism in response to cadmium in *Arabidopsis thaliana* and the related Zn/Cd-hyperaccumulator *Thlaspi caerulescens*. Plant Cell Environ..

[33] Durrett T.P., Gassmann W., Rogers E.E. (2007). The FRD3-mediated efflux of citrate into the root vasculature is necessary for efficient iron translocation. Plant Physiol..

[34] Broadley M.R., White P.J., Hammond J.P., Zelko I., Lux A. (2007). Zinc in plants. New Phytol..

[35] Shanmugam V., Lo J.C., Yeh K.C. (2013). Control of Zn Uptake in *Arabidopsis halleri*: A balance between Zn and Fe. Front. Plant Sci..

[36] Ivanov R., Bauer P. (2017). Sequence and coexpression analysis of iron-regulated ZIP transporter genes reveals crossing points between iron acquisition strategies in green algae and land plants. Plant Soil.

[37] Zheng X., Chen L., Li X. (2018). Arabidopsis and rice showed a distinct pattern in ZIPs genes expression profile in response to Cd stress. Bot. Stud..

[38] Sinclair S.A., Krämer U. (2012). The zinc homeostasis network of land plants. Biochim. Biophys. Acta.

[39] Zhang J., Martinoia E., Lee Y. (2018). Vacuolar transporters for cadmium and arsenic in plants and their applications in phytoremediation and crop development. Plant Cell Physiol..

[40] Qian Z. (2021). Analysis of Cadmium and Zinc Absorption Processes in A Hyperaccumulator *Arabidopsis halleri* ssp. *gemmifera*. Doctoral Dissertation.

[41] Emiliani J., Llatance Oyarce W.G., Bergara C.D., Salvatierra L.M., Novo L.A.B., Pérez L.M. (2020). Variations in the phytoremediation efficiency of metal-polluted water with *salvinia biloba*: Prospects and toxicological impacts. Water.

[42] Hothorn T., Bretz F., Westfall P. (2008). Simultaneous inference in general parametric models. Biom. J..

